# Growing Pains: The Search for Aging Studies Internships for Undergraduate Majors

**DOI:** 10.1093/geroni/igaf122.2946

**Published:** 2025-12-31

**Authors:** Nasreen Sadeq, Debra Dobbs, Lindsay Peterson, Sara Hackett, Lu Norstrand

**Affiliations:** University of South Florida, Tampa, Florida, United States; University of South Florida, Tampa, Florida, United States; University of South Florida, Tampa, Florida, United States; University of South Florida, Tampa, Florida, United States; University of South Florida, Tampa, Florida, United States

## Abstract

The School of Aging Studies at the University of South Florida recently completed a major curriculum revision of our B.S. in Health Care Administration program. Formerly the B.S. in Long-Term Care Administration, this undergraduate program was originally developed with guidance from the Florida Board of Nursing Home Administrators and remains the only program in Florida offering students the opportunity to complete a 9-credit (650 hour) Administrator in Training (AIT) internship. To increase student enrollment in our revised Health Care Administration major, we focused on emphasizing the opportunity to complete an internship as a key recruitment strategy. Our updated curriculum that allows students to choose between the AIT program or a 3-credit part-time internship resulted in 15 interns at 14 different internship sites in 2024, generating 4,850 service hours. We are expecting a two-fold increase in next year in the number of students needing an internship placement. Strategies implemented to improve the overall efficiency of the internship program will be presented, including the development of standard operating procedures, a database for tracking student progress, and online modules that provide students with an orientation to the internship program. Challenges we have experienced including the limited availability of internship sites and the extensive time and effort required to develop new community partnerships and to match students with gerontology-oriented internships aligned with their career goals will also be discussed. With the increasing trend of undergraduates seeking internships and field experiences, we will share our ongoing plans to continue addressing these challenges in the future.

